# Novel Intervention in the Aging Population: A Primary Meningococcal Vaccine Inducing Protective IgM Responses in Middle-Aged Adults

**DOI:** 10.3389/fimmu.2017.00817

**Published:** 2017-07-19

**Authors:** Marieke van der Heiden, Annemieke M. H. Boots, Axel A. Bonacic Marinovic, Lia G. H. de Rond, Marjan van Maurik, Irina Tcherniaeva, Guy A. M. Berbers, Anne-Marie Buisman

**Affiliations:** ^1^Centre for Infectious Disease Control (Cib), National Institute for Public Health and the Environment (RIVM), Bilthoven, Netherlands; ^2^Department of Rheumatology and Clinical Immunology, University of Groningen, University Medical Centre Groningen, Groningen, Netherlands

**Keywords:** immunosenescence, aging, middle-aged, primary immunization, IgM, *de novo* antigens, meningococci

## Abstract

**Introduction:**

Vaccine responses are often reduced in the elderly, leaving part of the elderly population vulnerable to infectious diseases. Timely vaccination may offer a solution for strengthening memory immunity before reaching old age, which classifies middle-aged persons as a target age group for vaccine interventions. However, knowledge regarding the immunogenicity of primary immunizations in middle-aged adults is lacking. We determined the immunogenicity of a primary meningococcal vaccine towards which no or (very) low pre-vaccination immunity exists in middle-aged adults (NTR4636).

**Methods:**

A vaccine containing multiple meningococcal groups (tetravalent) conjugated to tetanus toxoid (MenACWY-TT) was administered to middle-aged adults (50–65 years of age, *N* = 204) in a phase IV single-center and open-label study. Blood samples were taken pre-, 7 days, 28 days, and 1 year post-vaccination. Functional antibody titers were measured with the serum bactericidal assay (SBA). Meningococcal- and tetanus-specific antibody responses were determined with a fluorescent bead-based multiplex immunoassay. A bi-exponential decay model was used to estimate long-term protection.

**Results:**

In the majority of the participants, the meningococcal vaccine clearly induced naïve responses to meningococci W (MenW) and meningococci Y (MenY) as compared to a booster response to meningococci C (MenC). After 28 days, 94, 99, and 97% of the participants possessed a protective SBA titer for MenC, MenW, and MenY, respectively, which was maintained in 76, 94, and 86% 1 year post-vaccination. At this 1-year time point, significantly lower SBA titers were found in participants without a pre-vaccination SBA titer. Overall, protective antibody titers were predicted to persist after 10 years in 40–60% of the participants. The SBA titers correlated well with the meningococcal-specific IgM responses, especially for MenW and MenY. Interestingly, these IgM responses were negatively correlated with age.

**Conclusion:**

Primary immunization with a tetravalent meningococcal vaccine was highly immunogenic in middle-aged adults, inducing protective antibody titers in the vast majority of the participants lasting for at least 1 year. The age-related decrease in highly functional IgM responses argues in favor of vaccination against *de novo* antigens before reaching old age and, hence, middle-aged persons are an age group of interest for future vaccine interventions to protect the aging population.

## Introduction

The world population is rapidly aging. Worldwide predictions indicate that by the year 2050, the number of persons above 60 years of age is more than doubled as compared to 2015, with the numbers of persons above 80 years of age, the so-called “oldest-olds,” increasing even faster (1). Population aging has major medical implications, as with age vulnerability to both chronic and infectious diseases increases. Due to increased numbers of susceptible elderly, the population herd immunity against infectious diseases may diminish (2, 3). Therefore, prevention of infectious diseases by immunization of the elderly is a prerequisite for establishing healthy aging (4, 5).

With age, reduced thymic output of naïve T-cells and reduced bone marrow B-cell niches are observed, causing compositional changes in both compartments of the adaptive immune system (5–8). These alterations affect both the cellular and the humoral immune responses to vaccines (7, 9). For example, the elderly show reduced T-cell responses after varicella zoster vaccination (10), as well as reduced functional antibody responses after seasonal influenza, pneumococcal, and yellow fever vaccination (11–15). Larger effects of immunological aging are expected for *de novo* immune responses, as compared with the above-mentioned recall responses (hereafter called booster responses), due to reduced numbers of naïve cells (2, 3, 16).

Timely vaccination before reaching old age may offer a solution for circumventing these deleterious effects (2, 11). Due to the early appearance of the first signs of immune aging by the age of 50 (6), it has been suggested that immunization against new antigens most probably will have to be implemented at middle-age (2). However, the immunological fitness of middle-aged persons is largely unknown.

We determined the immunogenicity of a primary meningococcal vaccine in middle-aged adults (50–65 years of age). Due to herd immunity in the population after the mass vaccination campaign (in children 1–19 years of age) in 2002, circulation of meningococci C (MenC) is virtually non-existing, resulting in reduced memory immunity in the elderly population (17). Moreover, historical circulation of meningococci W (MenW) and meningococci Y (MenY) in the Netherlands has been very low, indicating that the meningococcal vaccination will most probably induce naïve type responses in older adults (17). Due to this expected low pre-vaccination immunity, the meningococcal vaccine was used as a model antigen to study primary vaccine responses in middle-aged adults.

Nowadays, next to young infants and adolescents, an increase of meningococcal cases is observed in persons over 65 years of age, which are mainly caused by MenW and MenY (18, 19). Also, the highest meningococcal case fatality rate has been reported in this age group, which may be due to underlying comorbidities (18–20). In addition, the currently ongoing MenW outbreak in the Netherlands is exactly following the course of the outbreak in the UK with 2 years delay, in which a large proportion of the invasive meningococcal disease cases was observed in persons above 65 years of age (21, 22). This vulnerability for meningococcal disease in the elderly population may further increase due to population aging (17, 23).

Until now, few studies investigated the immunogenicity of meningococcal vaccination in older adults (23, 24). In addition, immunogenicity studies were mostly performed in a historically naturally primed population. Here, we investigated the immunogenicity of a primary MenACWY-tetanus toxoid (TT) vaccination in an expected “immunologically naïve” middle-aged (50–65 years old) population. Moreover, a bi-exponential decay model was used to estimate the long-term protection induced by the vaccination in this middle-aged target group.

## Materials and Methods

### Study Design and Participants

Within this phase IV single-center and open-label study, invitations were sent to middle-aged adults (between 50 and 65 years of age) in Amersfoort, a city in the middle of the Netherlands, during August/September 2014. The Dutch community-based administration was used to send the invitations. Potential participants were excluded based on the following criteria: antibiotic use or fever (>38°C) within the last 14 days, serious diseases demanding immune suppressive medical treatment within the last 3 months, a known or suspected immune deficiency, a blood coagulation disorder, a neurologic disorder, administration of blood products in the past 6 months, serious surgery within the last 3 months, the use of hormone supplementation, pregnancy, a suspected allergy toward the vaccine components, history of serious adverse events after previous vaccinations, a previous meningococcal vaccination, a previous meningococcal episode, a tetanus vaccination within the last 5 years, and any vaccination in the month before enrollment. Written informed consent was obtained from all participants prior to enrollment and all procedures were in accordance with the Declaration of Helsinki. The medical ethical committee: Medical Research Ethics Committees United (MEC-U) approved the study and the study was registered at the Dutch trial register (NTR4636).

### Vaccination and Blood Sampling

A pre-vaccination blood sample was taken from all participants. Subsequently, all participants received the tetravalent meningococcal vaccine conjugated to TT (MenACWY-TT, Nimenrix, GlaxoSmithKline) by intramuscular administration. Blood samples were taken 7 days, 28 days, and 1 year post-vaccination. Serum samples were collected using serum clotting tubes (BD Biosciences) and were stored at −20°C until further use. All participants were sampled during evening hours. In addition, all participants filled in a short health questionnaire during the first appointment.

### Serological Analysis

IgG and IgG subclass concentrations specific for the polysaccharides of the meningococcal groups C, W, and Y (MenCWY), and TT were determined by the fluorescent-bead-based-multiplex immuno assay as previously described (25–28). An internationally accepted TT-specific IgG concentration of 0.01 IU/ml was used as protection level (29). A similar method was used to determine the MenCWY-specific IgM concentrations, with the modification of using a donkey anti-human IgM RPE conjugate (Fc5μ specific, Jackson ImmunoResearch).

MenA-specific measurements were left out of the analysis due to interference of varying levels of cross-reacting antibodies to MenA, present in these older age cohorts, in the immunoassay. Moreover, the analysis of MenCWY-specific responses is perceived sufficient to answer our primary research question in this study.

The MenCWY-specific protective antibody titers were determined with the serum bactericidal antibody assay using baby rabbit complement (rSBA) (Pelfreez, LOT#13035EL), as described previously (30, 31). The MenC, MenW, and MenY strains used in the rSBA were C11, MP01240070, and S-1975, respectively, kindly donated by Prof. Dr. Ray Borrow from the Vaccine Evaluation Unit at Manchester (PHE). The bactericidal titer was expressed as the reciprocal of the highest serum dilution yielding ≥50% killing after 60 min of incubation. The internationally accepted correlate of protection used was an rSBA titer of ≥8 for all groups, whereas a titer of ≥128 was used as a more conservative measure for long-term protection (31, 32). Participants with an rSBA titer below detection level (rSBA seronegative) were assigned an rSBA titer of two for statistical purposes. A representative group of 100 persons was selected for the functional rSBA analysis, based on varying IgG levels from low to high concentrations, for all three meningococcal groups. For MenW and MenY, the same selection of participants was used. This selection had an overlap of 71 participants with the selection for MenC.

Gullsorb reagent human IgG (Meridian Biosciences™) was used for IgG depletion and goat-Anti-human IgM (μ-chain specific)-Agarose antibody beads (Sigma Aldrich) for IgM depletion in the serum samples.

### Statistics

Normal distribution of the data was checked prior to each analysis and only cases with samples available at all time points were included in the analysis. Geometric mean concentrations (GMCs) with 95% confidence intervals [95% CI] were calculated for the MenCWY-PS- and TT-specific IgG responses, as well as the MenCWY-PS-specific IgG subclass and IgM responses. MenCWY-PS-specific IgG and IgM concentrations were log transformed, after which the repeated measurements ANOVA was used to analyze the post-vaccination response. rSBA geometric mean titers (GMTs) with the corresponding 95% CI were calculated. Proportions and 95% CI of participants with an rSBA ≥8 and ≥128 were calculated with the Wilson/Brown test. The proportion of participants with an rSBA ≥8 and ≥128 were compared between participants with and without a pre-vaccination serum bactericidal assay (SBA) titer with the Chi-squared test, whereas differences in GMTs were analyzed with the Mann–Whitney *U* test. Differences in IgG ratios between the meningococcal groups were analyzed with the Mann–Whitney *U* test preceded by the Kruskal–Wallis test and corrected for multiple comparisons with the Bonferroni correction.

Correlations between the rSBA titers and the IgG and IgM responses 28 days and 1 year post-vaccination were determined using the Spearman’s rho correlation test. Graphpad Prism V7 and SPSS V22.0 were used for the statistical analysis. A *p*-value <0.05 was considered statistically significant.

### Bi-Exponential Decay Model

To study the duration of protection, we used a Bayesian approach with a multilevel longitudinal model to statistically predict the dynamics of antibody concentrations (33). We performed Markov-chain Monte Carlo simulations to find the appropriate joint distribution of parameters which best could adjust the model to the measured rSBA titer datasets for MenC, MenW, and MenY. Assuming a simple exponential decay after reaching the time to peak (*t*_1_) would underestimate the persistence of protection (33–35). Therefore, we modeled the rSBA titer decay assuming that it follows a bi-exponential decay curve of the form:

$$y(t>t_{1})=y_{1}\left(e^{-\alpha_{1}\left(t-t_{1}\right)}+fe^{-\alpha_{2}\left(t-t_{1}\right)}\right)/(1+f)$$

where *y*_1_ is the peak antibody concentration level, α_1_ and α_2_ are the respective decay rates of each exponential component, and *f* regulates the contribution of the exponential components.

The initial raise of antibody levels was assumed to be exponential until the time to peak *t*_1_. Per individual, the three measured antibody levels were used to fit the multilevel model with their respective time point. The time to peak (*t*_1_) in the model was set to follow a lognormal distribution with a mean of 12 days (SD 8.5 days). The long-term antibody persistence was described using the decay curves predicted by the models. To assess the duration of protection, we focused on the predicted percentages of participants that possessed an rSBA titer >8 and >128, for any given time up to 10 years post-vaccination.

## Results

### Study Population

**Figure 1:**
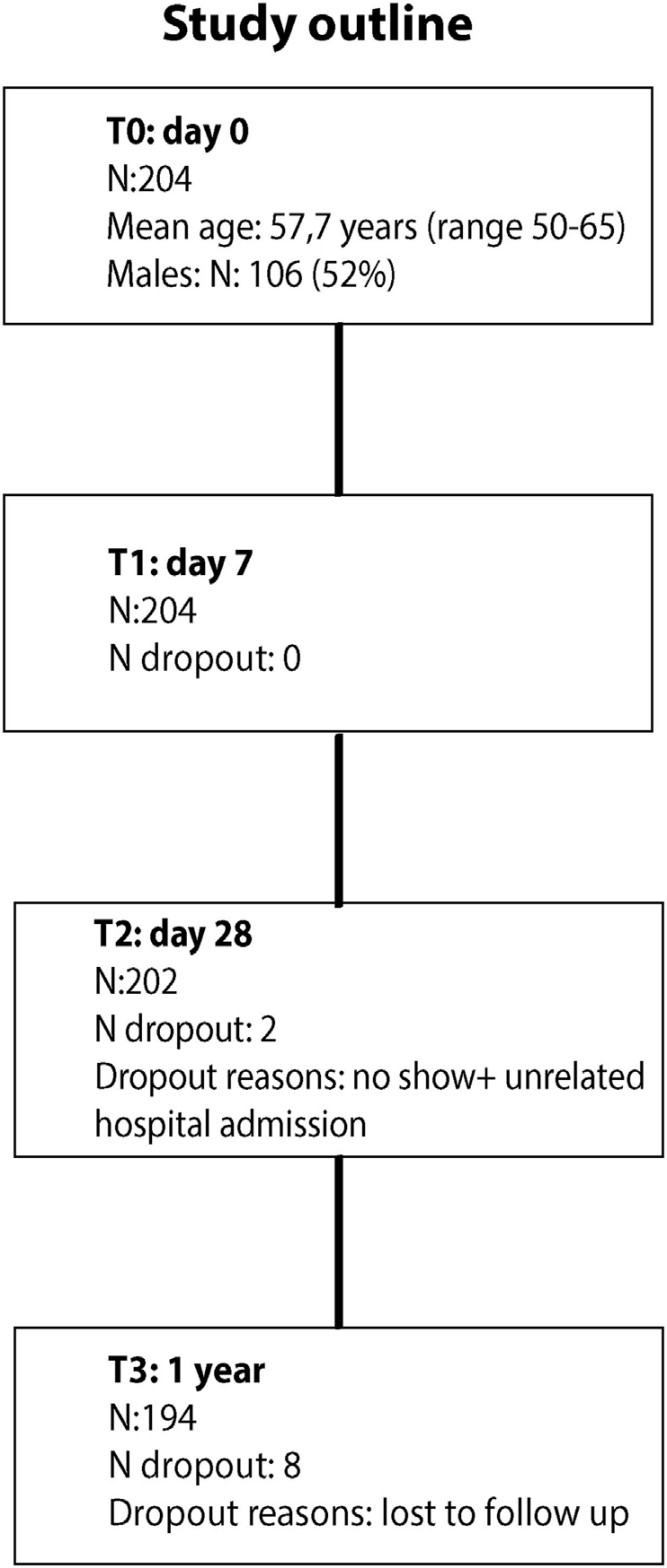
Participant flow chart.

A total of 204 middle-aged adults (mean age: 57.7 years; range 50–65 years; 52% males) participated in the study (Figure 1). All participants received the MenACWY-TT vaccine. Overall, 194 participants (95.1%) completed the study, with blood drawings before vaccination and at 7, 28 days, and 1 year post-vaccination. Additional baseline participant characteristics are presented in Table S1 in Supplementary Material.

### IgG Responses Reflect General Naïve and Booster Responses after MenACWY-TT Vaccination in Middle-Aged Adults

**Figure 2:**
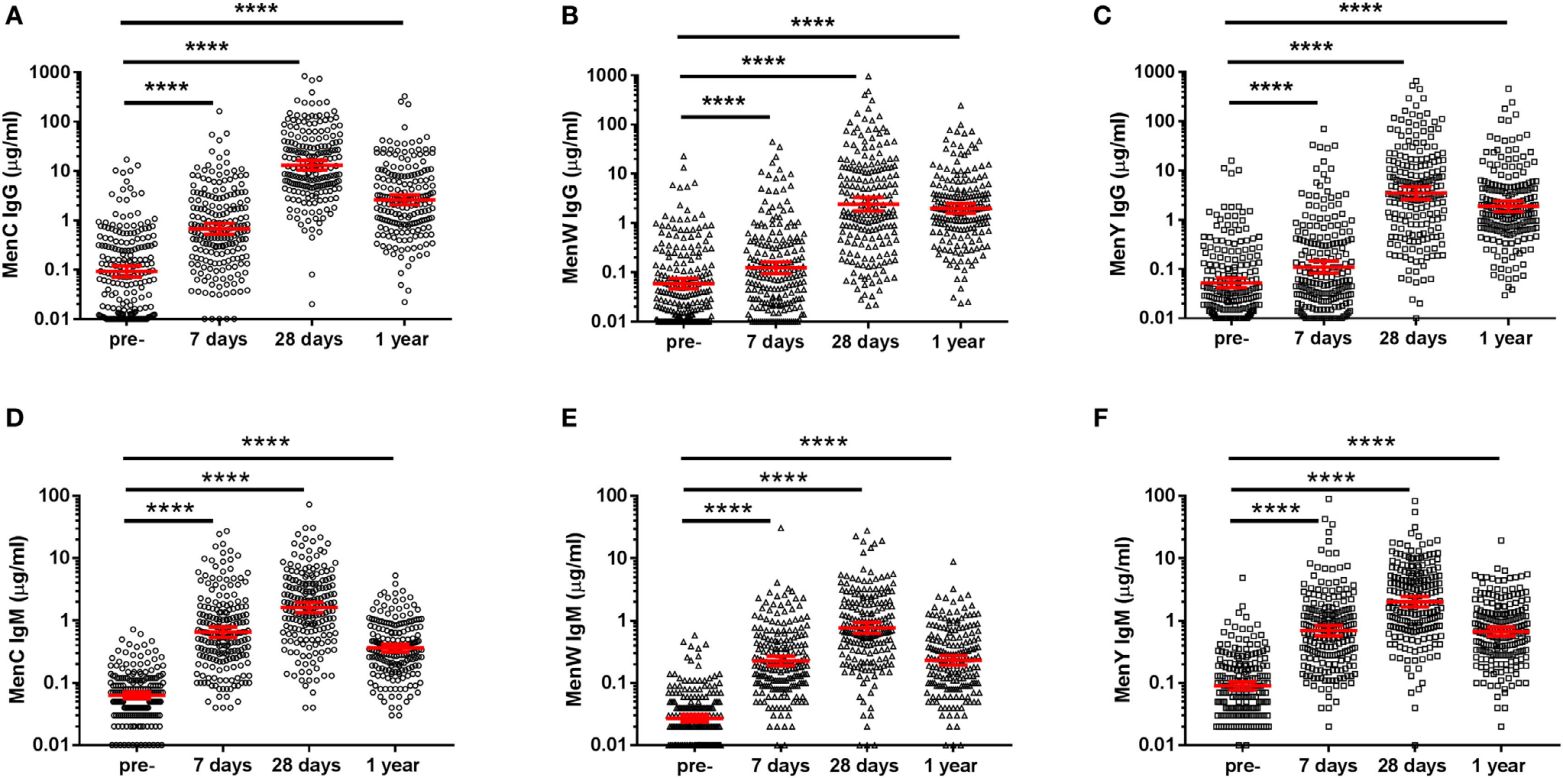
MenCWY-specific IgG and IgM responses. MenC **(A)**, MenW **(B)**, MenY **(C)** polysaccharide-specific IgG, and MenC **(D)**, MenW **(E)**, MenY **(F)** polysaccharide-specific IgM responses pre- and post-vaccination. The lines indicate the geometric mean concentrations with 95% CI intervals. The different time points were compared with the repeated measured ANOVA, which was highly significant for all comparisons (*p* < 0.0001), after which pairwise comparisons between the time points were performed.

At first, longitudinal MenCWY and TT-specific IgG responses were investigated (Figures 2A–C; Figure S1 in Supplementary Material). Pre-vaccination, low IgG levels were observed for the different meningococcal groups. Seven days post-vaccination, IgG responses were enhanced for all three groups (Figures 2A–C), although a higher increase in the IgG response (ratio day 7/pre) was observed for MenC as compared to MenW and MenY (Table 1). This was also reflected in the number of persons with an IgG fold increase above four at day 7, which was 58.8, 20.1, and 22.5% for MenC, MenW, and MenY, respectively (Table 1). Moreover, compared to the other groups, a significant higher increase in MenC-specific IgG was observed 28 days post-vaccination (ratio day 28/pre) (Table 1). Taken together, these results are suggestive of a booster response to MenC as compared to a naïve response in the majority of the participants to MenW and MenY. Nonetheless, based on the IgG response 7 days post-vaccination, a few participants also showed booster responses for MenW and MenY as well as naïve responses to MenC. In addition, MenC-specific IgG concentrations showed a larger decay from 28 days to 1 year (ratio day 28/1 year) post-vaccination than seen for MenW and MenY IgG levels, resulting in comparable IgG GMCs 1 year post-vaccination for all three groups (Table 1). Notably, these IgG concentrations were still significantly enhanced compared with pre-vaccination levels (Figures 2A–C). The TT conjugate clearly induced a booster response in the participants, since 71.6% of the persons showed highly significant increases in IgG concentrations 7 days post-vaccination (Table 1). In the majority of the middle-aged persons, pre-vaccination TT-specific IgG concentrations were above the protection level of 0.01 IU/ml and these were significantly enhanced up to 1 year post-vaccination (Table 1; Figure S1 in Supplementary Material). Within this cohort, we did not observe any effects of age, gender, and CMV seropositivity on the IgG responses (Table S2 in Supplementary Material).

**Table 1:** MenCWY and tetanus toxoid (TT)-specific IgG responses.

	MenC (μg/ml)	MenW (μg/ml)	MenY (μg/ml)	TT (IU/ml)
Pre-GMC [95% CI]	0.09 [0.06–0.11]	0.06 [0.04–0.07]	0.05 [0.04–0.06]	0.76 [0.63–0.91]
Day 7 GMC [95% CI]	0.67 [0.52–0.86]	0.13 [0.09–0.16]	0.11 [0.08–0.14]	6.81 [5.84–7.94]
Ratio day 7/pre [95% CI]	7.3 [5.9–9.0]	2.1 [1.7–2.4]****^,^^a^	2.1 [1.8–2.5]****^,^^a^	8.6 [7.4–10.6]
Number of fold change > 4 (%)	120 (58.8%)	41 (20.1%)****^,^^a^	46 (22.5%)****^,^^a^	146 (71.6%)
Day 28 GMC [95% CI]	13.03 [10.39–16.35]	2.43 [1.78–3.33]	3.49 [2.59–4.71]	13.89 [11.71–16.48]
Ratio day 28/pre [95% CI]	141 [107.5–185.1]	41.6 [31.4–55.1]****^,^^a^^/^*^,^^b^	67.9 [52.2–88.2]**^,^^a^	18.5 [15.5–22.1]
1 year GMC [95% CI]	2.60 [2.06–3.28]	1.99 [1.59–2.51]	1.89 [1.48–2.41]	3.51 [3.03–4.07]
Ratio day 28/1 year [95% CI]	5.0 [4.4–5.6]	1.3 [1.1–1.5]****^,^^a^	1.8 [1.6–2.2]****^,^^a^	3.9 [3.5–4.3]

*The MenC, MenW, and MenY polysaccharide and TT-specific geometric mean concentrations (GMCs [95% CI], in microgram per milliliter) at the different time points pre- and post-vaccination. Moreover, ratios between day 7/pre, day 28/pre, day 28/1 year, and the number of persons with an IgG fold change above four at day 7 are indicated for the different groups*.

*p < 0.05, **p < 0.01, ****p < 0.0001.

*^a^Compared to MenC*.

*^b^Compared to MenY*.

*The MenC, MenW, and MenY-specific ratios at day 7, 28, and 1 year were compared with the Mann–Whitney U test after correction for multiple testing. Moreover, the numbers of persons with a fold change >4 at day 7 were compared with the Chi-square test*.

Remarkably, a robust IgM response was observed for all meningococcal groups and these IgM levels were still enhanced 1 year post-vaccination (Figures 2D–F). A significant negative correlation was found between the IgM responses and age for MenC and MenW, whereas a negative trend was observed for MenY (Table 2). However, the low predictive value of the model (R^2^ model) indicates that age and pre-existing IgM concentrations were not the major factors predicting the IgM concentrations post-vaccination.

**Table 2:** Effect of age and gender on the MenCWY IgM responses.

Group	Timepoint	Predicting variable	*p*-Value	β coefficient	*R*^2^ model
MenC	7 days	**Pre-IgM**	**0.002**	**0.218**	0.102
		**Age**	**0.001**	**−0.237**	
		Gender	0.740	−0.022	
	28 days	Pre-IgM	0.046	0.142	0.048
		Age	0.018	−0.168	
		Gender	0.158	−0.100	
	1 year	**Pre-IgM**	**0.000**	**0.292**	0.106
		Age	0.024	−0.158	
		Gender	0.426	−0.055	
MenW	7 days	**Pre-IgM**	**0.000**	**0.246**	0.154
		**Age**	**0.000**	**−0.315**	
		Gender	0.326	−0.066	
	28 days	Pre-IgM	0.111	0.115	0.055
		**Age**	**0.002**	**−0.215**	
		Gender	0.256	−0.081	
	1 year	**Pre-IgM**	**0.000**	**0.307**	0.148
		Age	0.010	−0.176	
		Gender	0.045	−0.139	
MenY	7 days	**Pre-IgM**	**0.001**	**0.238**	0.090
		Age	0.019	−0.161	
		Gender	0.074	−0.123	
	28 days	Pre-IgM	0.554	0.043	0.030
		Age	0.037	−0.148	
		Gender	0.067	−0.131	
	1 year	**Pre-IgM**	**0.000**	**0.272**	0.100
		Age	0.108	−0.113	
		Gender	0.049	−0.139	

*Linear regression was performed using the log-transformed IgM concentrations. Age was included as a continuous variable. Gender: 0 = female, 1 = male. The effects of age and gender were adjusted for the presence of pre-vaccination immunity (Pre-IgM), since pre-IgM was perceived a confounder. After correction for multiple testing, a *p*-value <0.05/9 = 0.006 was considered significant. Significant correlations are indicated in bold*.

### The Meningococcal Groups Induced Protective rSBA Responses in Middle-Aged Adults

A representative group of 100 persons was selected for the functional rSBA analysis, based on the IgG concentrations. As expected, the majority of the participants possessed pre-vaccination rSBA titers below the internationally accepted protection level of 8. MenC, MenW, and MenY-specific pre-vaccination rSBA titers ≥8 were observed in 18, 23, and 27% of the middle-aged participants (Table 3). Moreover, 28 days post-vaccination, the vast majority of the participants possessed an rSBA titer ≥8, which was 94, 99, and 97% for MenC, MenW, and MenY, respectively. In addition, 92, 97, and 95% for MenC, MenW, and MenY, respectively, possessed an rSBA titer ≥128, the cutoff titer used for long-term protection (Table 3). One-year post-vaccination, protective antibody titers were still found in 76–94% of the participants (Table 3). Surprisingly, at this time point, slightly higher rSBA titers were observed for MenW and MenY compared with MenC. Moreover, a small, but significant negative correlation of the rSBA titer was found with age; 28 days post-vaccination for MenC (rho: −0.239, *p*: 0.017) and 1 year post-vaccination for MenW (rho: −0.300, *p*: 0.002) (Table S3 in Supplementary Material). No gender differences were observed (data not shown).

**Table 3:** MenCWY-specific serum bactericidal antibody responses using baby rabbit complement (rSBA).

Time point	Value	MenC	MenW	MenY
Pre-	GMT	3.9 [2.9–5.3]	5.4 [3.8–7.9]	6.9 [4.5–10.4]
	≥8%	18 [11.7–26.7]	23 [15.8–32.2]	27 [19.3–36.4]
	≥128%	7 [3.4–13.8]	8 [4.1–15.0]	17 [10.9–25.5]
28 days	GMT	1,469 [950.5–2,269]	1,771 [1,354–2,315]	1,448 [1,026–2,044]
	≥8%	94 [87.5–97.2]	99 [94.6–100]	97 [91.5–99.2]
	≥128%	92 [85.0–95.9]	97 [91.5–99.2]	95 [88.8–97.8]
1 year	GMT	111.4 [66.6–186.4]	335.5 [236.9–475.1]	247.3 [158.1–386.7]
	≥8%	76 [66.8–83.3]	94 [87.5–97.2]	86 [77.9–91.5]
	≥128%	61 [51.2–70]	74 [64.6–81.6]	79 [70.0–85.8]

*MenCWY-specific GMTs are presented with the [95% CI]. The percentages and [95% CI] of participants with an rSBA titers above 8 (cutoff for protection) and 128 (cutoff for long-term protection) are given at all time points*.

*GMT, Geometric mean titer*.

### Modeling of the Protection Levels 10 Years Post-Vaccination

Using a bi-exponential decay model, the proportion of participants with rSBA titers above the protective cutoff level of 8 at 10 years post-vaccination was estimated to be around 40, 40, and 60% for MenC, MenW, and MenY, respectively (Figures S2A–C in Supplementary Material). Moreover, 20, 20, and 40% of the participants were expected to possess rSBA titers above the protective cutoff level of 128 at 10 years post-vaccination for MenC, MenW, MenY, respectively (Figures S2D–F in Supplementary Material).

### Differences in rSBA Response between Participants with and without Pre-Vaccination rSBA Titers

**Figure 3:**
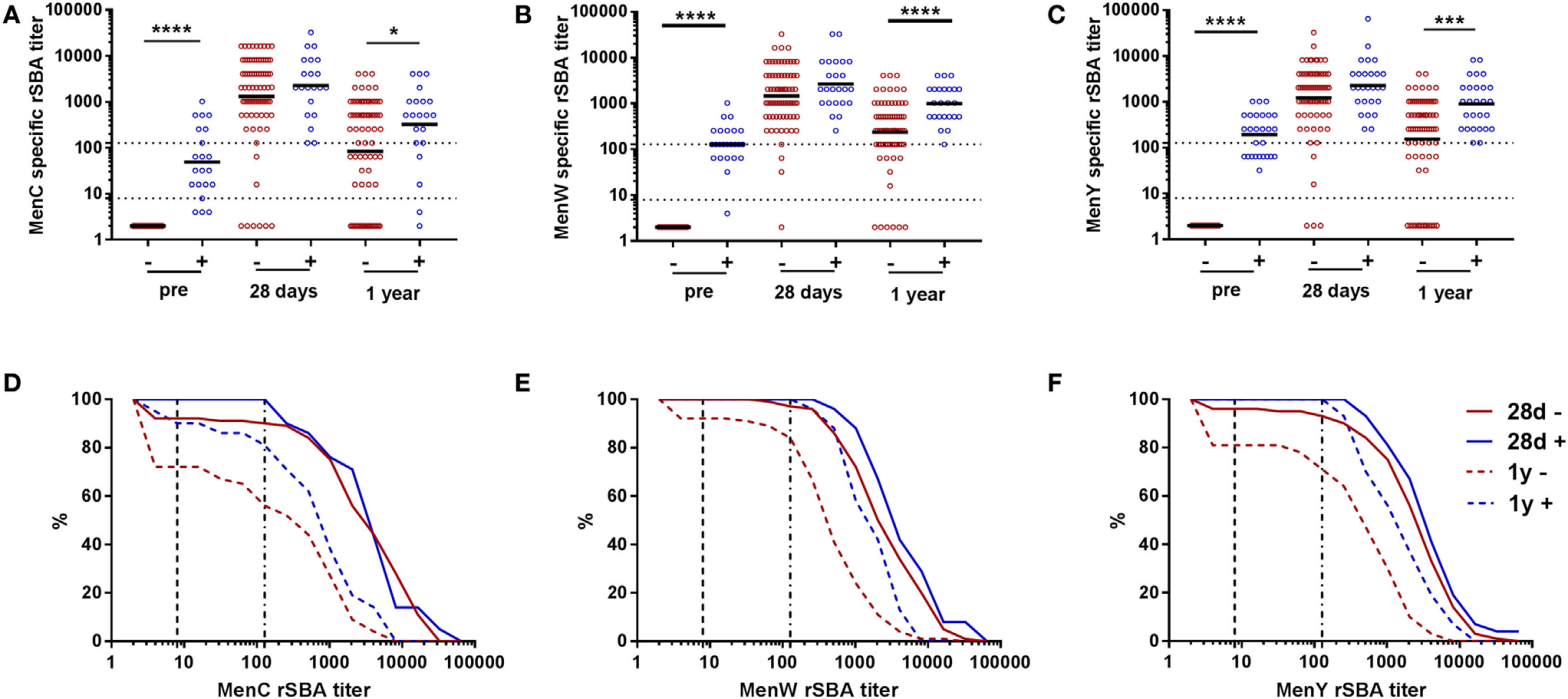
MenCWY serum bactericidal antibody responses in participants with and without pre-vaccination rSBA titers. MenC **(A)**, MenW **(B)**, and MenY **(C)** specific rSBA responses at the different time points for pre-vaccination seronegative (−, red) and seropositive (+, blue) participants. The geometric mean titers are indicated. The seronegative and seropositive participants were compared at the different time points using the Mann–Whitney *U* test *p < 0.05, ***p < 0.001, and ****p < 0.0001. Reverse cumulative distribution graphs at day 28 and 1 year post-vaccination for MenC **(D)**, MenW **(E)**, and MenY **(F)** separated for the pre-vaccination seronegative (red) and seropositive (blue) participants.

In one-fourth of the participants, a detectable pre-vaccination rSBA titer (rSBA ≥ 4) was observed (Figures 3A–C). Subsequently, we compared the vaccine response between participants being seropositive (rSBA ≥ 4) or being seronegative at the pre-vaccination time point (Figure 3; Table S4 in Supplementary Material). Similar rSBA titers were observed in both groups 28 days post-vaccination for all meningococcal groups (Figures 3A–C; Table S4 in Supplementary Material), indicating a robust increase in rSBA titer in the seronegative participants. At day 28, 89.9, 96.1, and 93.2% of the seronegative participants showed an rSBA titer ≥128 for MenC, MenW, and MenY, respectively, compared with 100% of the seropositive participants (Figures 3D–F; Table S4 in Supplementary Material). One-year post-vaccination, rSBA titers showed a significantly higher decay in the seronegative participants, reaching significance for MenW and MenY, and resulting in rSBA titers below the protection limit (rSBA ≥ 8) in some participants (Figure 3; Table S4 in Supplementary Material).

### Strong Correlations between the MenW- and MenY-Specific IgM Responses and Antibody Functionality

**Figure 4:**
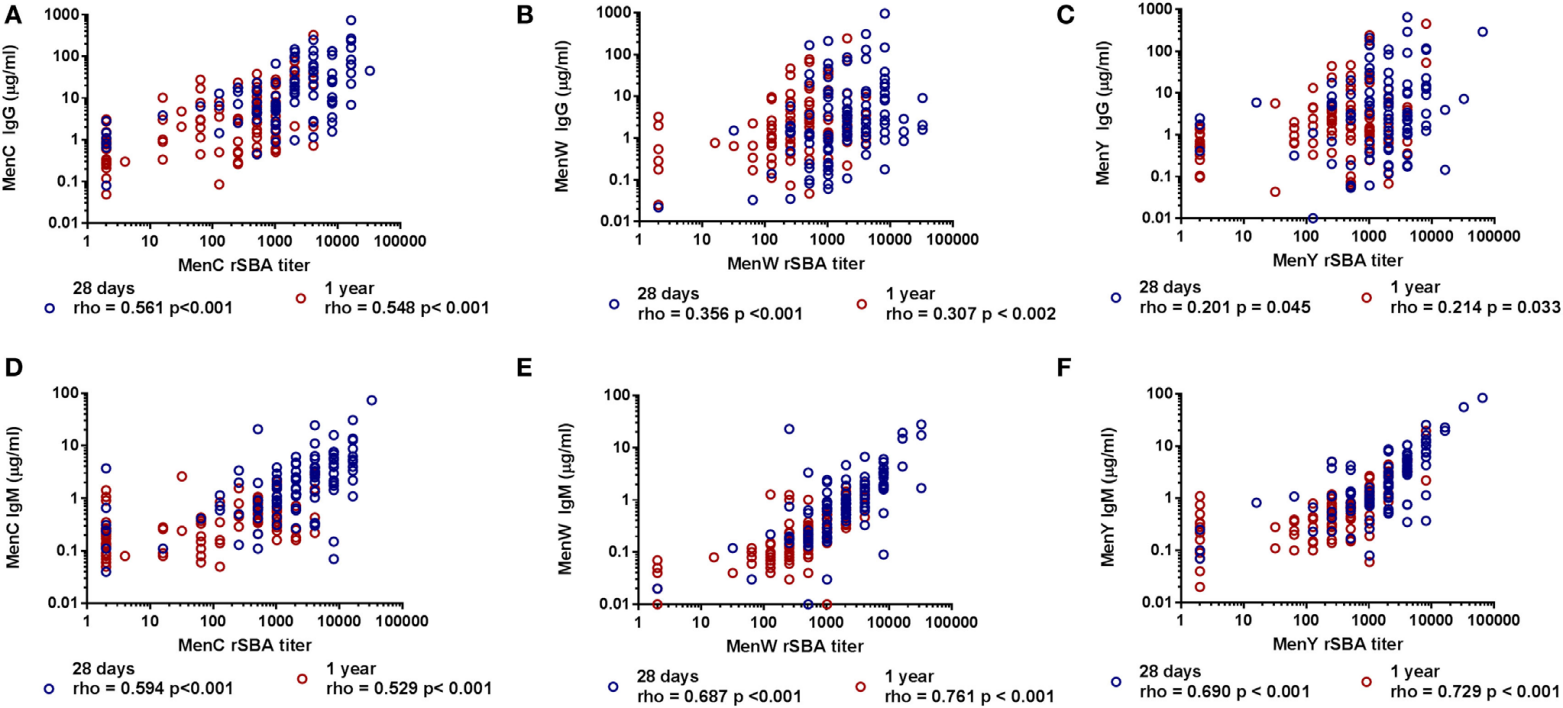
Correlation between the MenCWY-specific IgG and IgM responses with the serum bactericidal antibody responses. The correlation between the MenC **(A)**, MenW **(B)**, and MenY **(C)** PS-specific IgG responses and the MenC **(D)**, MenW **(E)**, and MenY **(F)** PS-specific IgM responses with the rSBA responses at 28 days and 1 year post-vaccination. Correlations were determined with the Spearman’s rho correlation test.

Since the rSBA titers and IgG responses for the different groups did not show a similar pattern, the correlations between the IgG and rSBA responses for the three meningococcal groups were determined (Figures 4A–C). A moderate correlation was found for MenC (28 days: rho 0.561, *p* < 0.001, 1 year: rho 0.548, *p* < 0.001) (Figure 4A), whereas the correlations for MenW (28 days: rho 0.356, *p* < 0.001, 1 year: rho 0.307, *p* < 0.002) and MenY (28 days: rho 0.201, *p* = 0.045, 1 year: rho 0.214, *p* = 0.033) were rather low (Figures 4B,C).

This encouraged us to investigate whether MenC-, MenW-, and MenY-specific IgM responses could explain this low correlation. Remarkably, a good correlation was found between the rSBA titers and the IgM responses for both MenW (28 days: rho 0.687, *p* < 0.001, 1 year: rho 0.761, *p* < 0.001) and MenY (28 days: rho 0.690, *p* < 0.001, 1 year: rho 0.729, *p* < 0.001) (Figures 4E,F), whereas a moderate correlation was found for MenC (28 days: rho 0.594, *p* < 0.001, 1 year: rho 0.529, *p* < 0.001) (Figure 4D). In order to confirm the important contribution of the IgM antibodies to the rSBA response, IgG was depleted in a subset of the samples varying in SBA titer (Figures S3A,C in Supplementary Material). MenW and MenY rSBA titers remained almost equal after IgG removal. By contrast, these rSBA titers were largely reduced after IgM depletion (Figures S3B,D in Supplementary Material), although the rSBA titer was mostly retained in participants with very high IgG concentrations. The data suggest that the protective rSBA titers are mediated via induction of IgM type antibodies, mainly for MenW and MenY.

### Differential Meningococcal Group-Specific IgG Subclass Responses

Finally, we investigated whether IgG subclass responses differed between the naïve (MenW and MenY) and booster (MenC) responses at middle-age, as IgG subclass responses have previously been shown to affect the antibody functionality. IgG subclass responses were highly variable for all three groups. A slightly skewed IgG2 response was observed for MenC and MenW, whereas the response to MenY showed IgG1 skewing, as shown by the IgG1/IgG2 ratio (Table 4). Besides a primarily MenC-specific IgG2 skewed response, 1 year post-vaccination IgG1 and IgG2 contributed equally to the IgG response, suggesting a more rapid decay of IgG2 than IgG1 over time (Table 4), which was not seen for the IgG2 skewed MenW response. Also, the IgG1 skewed response to MenY remained stable.

**Table 4:** MenCWY-specific IgG subclass responses.

Timepoint	Measurement	MenC (μg/ml)	MenW (μg/ml)	MenY (μg/ml)
28 days	IgG1 [95% CI]	3.1 [2.53–3.85]	0.4 [0.30–0.52]	2.3 [1.80–2.83]
	IgG2 [95% CI]	5.1 [3.84–6.67]***^,^^a^	0.7 [0.49–1.03]***^,^^a^	1.4 [0.97–1.92]**^,^^a^
	IgG1/IgG2 ratio [95% CI]	0.6 [0.48–0.81]	0.6 [0.40–0.76]	1.6 [1.19–2.21]
1 year	IgG1 [95% CI]	1.0 [0.82–1.16]	0.6 [0.40–0.76]	1.6 [1.30–1.98]
	IgG2 [95% CI]	1.2 [0.85–1.47]	0.8 [0.63–1.11]**^,^^b^	0.8 [0.62–1.13]***^,^^b^
	IgG1/IgG2 ratio [95% CI]	0.8 [0.65–1.10]	0.7 [0.50–0.87]	1.9 [1.41–2.51]

*Geometric mean concentrations [95% CI] are indicated. Meningococcal group-specific IgG1 and IgG2 responses at the different time points were compared with the Mann–Whitney *U* test*.

**p < 0.01, ***p < 0.001.

*^a^IgG2 vs IgG1 at day 28*.

*^b^IgG2 vs IgG1 at 1 year*.

## Discussion

In this study, we demonstrate that a primary tetravalent meningococcal vaccine conjugated to TT (MenACWY-TT) in middle-aged adults is highly immunogenic. The vast majority (94–99%) of the participants developed protective antibody titers against three meningococcal groups 1 month post-vaccination. Moreover, 1 year post-vaccination protective antibody titers were still found in 76–94% of the participants. This level of protection was slightly lower in participants without detectable pre-vaccination rSBA titers compared with participants with pre-vaccination rSBA titers. Overall, in about 40–60% of the participants protective antibody titers are predicted to last for 10 years post-vaccination. Of importance, the protective rSBA titers were strongly associated with the meningococcal group-specific IgM responses, especially for MenW and MenY. Remarkably, these IgM responses were still enhanced 1-year post-vaccination and declined with advancing age even in this middle-aged (50–65 years) group.

Since our study is one of the few evaluating the immunogenicity of the MenACWY-TT vaccine in older adults, comparative data are scarce. We observed similar proportions of participants with protective post-vaccination titers (rSBA > 128) 1 month post-vaccination as reported by a study evaluating the immunogenicity of the MenACWY-TT vaccination in persons 56–103 years of age, of which 67% was middle-aged (56–65), in Lebanon (23). The Lebanese population, however, has a different epidemiological background than the Dutch population, since high meningococcal pre-vaccination levels were found in most Lebanese adults (23). Despite these differences in pre-vaccination immunity, comparable short-term vaccine responses were observed. In addition, two studies in the USA reported the immunogenicity of a pneumococcal conjugate vaccine with a similar composition of conjugated polysaccharides (the PCV13 vaccine), in older adults (50–69 years of age) 1 year post-vaccination (36, 37). However, since a large part of the older adult populations is likely to be primed naturally for the majority of the pneumococcal groups, these vaccines induced a booster response. In our study, a large induction of MenC-specific IgG concentrations 7 days post-vaccination was indicative of a booster response for MenC in most participants (38, 39). Since this meningococcal group induced robust functional antibody responses as measured by the SBA in our study adults, we confirmed the immunogenicity of a booster vaccine in middle-aged adults. In addition, we demonstrated robust antibody responses against the groups MenW and MenY, which showed a more *de novo* immune response in the majority of the middle-aged adults.

The observations of booster responses for MenC in the majority of the middle-aged adults, and the more naïve responses for MenW and MenY are in agreement with the meningococcal circulation in the Dutch population in the past (17). The MenC-specific booster response suggests long-term persistence of MenC-specific IgG-based memory immunity in adults, since the circulation of MenC was nearly eradicated after the mass vaccination campaign of 2002 (40). These memory cells may reside in the bone marrow and do not necessarily correlate with serum antibody levels (41, 42). The currently ongoing circulation of MenW and MenY might explain the high pre-vaccination SBA titers and IgM concentrations that were found in a small part of the participants, suggesting prior exposure (18, 21). However, meningococcal carriage studies in older adults are lacking to confirm our results.

We observed highly variable IgG subclass responses in the middle-aged adults for all meningococcal groups, a common finding after conjugate-carrier vaccination in adults (43–45). This may be explained by multifactorial causes such as host-specific contacts during life, chemical antigen characteristics as well as the age of the vaccinees (45, 46). Clearly, this would require further study. In this study, MenC- and MenW-specific IgG responses showed a skewing toward the IgG2 subclass, whereas IgG1 skewing was seen for MenY. Since, in general, IgG1 shows better complement binding compared with IgG2 (47), the MenY-specific IgG1 skewed response could be considered to be more functional. However, we found the weakest correlation between the total IgG concentrations and rSBA titers for MenY, which does not support the higher functionality of the MenY-specific IgG response in bacterial killing. Therefore, in adults, the exact roles of the IgG subclass responses in antibody functionality remains incompletely understood.

Remarkably, rSBA titers were largely determined by the group-specific IgM response, especially for MenW and MenY, since depletion of IgG did not significantly reduce the rSBA response, whereas IgM depletion did. IgM is known to be highly effective in complement binding (48) and found to be essential in the functionality of the pneumococcal antibody response (12). We here observed a negative correlation between the IgM response post-meningococcal vaccination and age in middle-aged adults, within a relatively small age range of 15 years. Therefore, we do expect a reduced functional meningococcal antibody response after primary immunization or even infection in the elderly. This expectation is strengthened by previous observations of an inverse correlation between serum IgM as well as IgM + B-cells and age (12, 46, 49–51). It may be possible to extrapolate to other primary bacterial infections and vaccinations in the elderly. Therefore, we propose to measure IgM responses, next to IgG, following primary bacterial vaccinations in middle-aged and elderly populations. Remarkably, participants possessing very high pre-vaccination IgG concentrations tended to show lower IgM concentrations post-vaccination and did not show a decrease in rSBA titer after IgM depletion. This finding suggests that the antibody functionality is reliant on high levels of IgG during clear booster responses. Since IgG responses may be less affected during the aging process, functional antibody responses develop after additional booster vaccinations during old age. Furthermore, unlike often reported for other pathogens (52), no clear gender differences were observed in the vaccine response in this age group.

In addition, we show high pre-vaccination TT-specific IgG concentrations in the majority of the middle-aged adults, which were highly boosted by the MenACWY-TT vaccination. This finding is different to the study in the Lebanese population, who possessed very low pre-vaccination TT-specific antibody levels that were only minimally boosted by the vaccination (23). This difference in pre-vaccination immunity may be caused by higher frequencies of TT booster vaccinations in the Dutch adults due to traveling or by higher frequencies of childhood TT vaccinations in the Netherlands. Previous surveillance studies also revealed relatively high TT antibodies in Dutch older adults (53). Since the TT carrier protein in this conjugated meningococcal vaccine is added to induce T-cell help in response to the meningococcal polysaccharides in order to induce long-term immunity and memory B-cell formation (54), the amount of T-cell help in our study may be different from the Lebanese population.

This study has important strengths, such as the use of a multivalent vaccine, containing antigens that induced both booster and naïve responses in the same participant. This allowed us to compare the immunogenicity of naïve and booster responses within the same group of middle-aged adults. Moreover, the blood sampling at 7 days post-vaccination strengthened our assumption of a general booster response for MenC and more naïve responses for MenW and MenY. In addition, the vaccine immunogenicity was based on an internationally accepted protective threshold in antibody functionality. Although we only measured this antibody functionality in a selection of 100 participants, this selection was representative for the entire cohort, since two independent selections of 50 participants, based on varying IgG responses from low to high concentrations, showed similar rSBA results. Finally, our data add clinically relevant information about the immunogenicity of the MenACWY-TT vaccine in middle-aged adults, which could be used to cease the currently ongoing MenW outbreak or to decrease the vulnerability of the future elderly population for new outbreaks. However, our study was limited in predicting long-term protection due to the short follow-up period. Using the bi-exponential decay model, we estimate that about 40–60% of our cohort may still be protected 10 years post-vaccination. However, this model needs to be validated by additional sampling of the participants several years post-vaccination. These additional samples are essential to determine the exact beneficial effects of vaccination at middle age for upholding memory immunity in the elderly.

To conclude, primary immunization with a tetravalent meningococcal vaccine, which contains antigens for which no or (very) low pre-vaccination immunity exists, was highly immunogenic in middle-aged adults. One-year post-vaccination protective antibody titers were still found in the vast majority of the participants. Future follow-up studies can determine the long-term protection of this meningococcal vaccination in elderly participants, although long-term protection is predicted in about half of the participants using bi-exponential decay modeling. Moreover, IgM was found essential in the antibody functionality against the new antigens and showed a decrease with advancing age. These findings support the suggestion that immunization against *de novo* antigens should be implemented before reaching old age. In short, our results imply that enhancing immunological memory by primary vaccination of middle-aged persons is feasible and provides a basis for novel strategies to extend protective immunity until old age.

## Ethics Statement

This study was carried out in accordance with the recommendations of the Medical Research Ethics Committees United (MEC-U) with written informed consent from all subjects. All subjects gave written informed consent in accordance with the Declaration of Helsinki. The protocol was approved by the Medical Research Ethics Committees United (MEC-U) and registered in the Dutch trail register (NTR4636).

## Author Contributions

MH, AB, GB, and A-MB designed the experiments. MH and LR planned and performed the clinical work. MH, LR, MM, and IT executed the laboratory experiments. AM performed the bi-exponential modeling. MH, AB, GB, and A-MB analyzed and interpreted the data. MH, AB, AM, GB, and A-MB wrote the manuscript. All authors critically revised the manuscript.

## Conflict of Interest Statement

MH, AM, LR, MM, IT, GB, and A-MB declare no conflict of interest. AB is a consultant for Grunenthal Gmbh (Germany).
