# Supplementary material for: Novel Intervention in the Aging Population: A Primary Meningococcal Vaccine Inducing Protective IgM Responses in Middle-Aged Adults
Source: Front Immunol. 2017 Jul 19;8:817. doi: 10.3389/fimmu.2017.00817 (PMC5515833; doi:10.3389/fimmu.2017.00817)
Supplement: Supplementary file 2 [file Image_1.PDF]

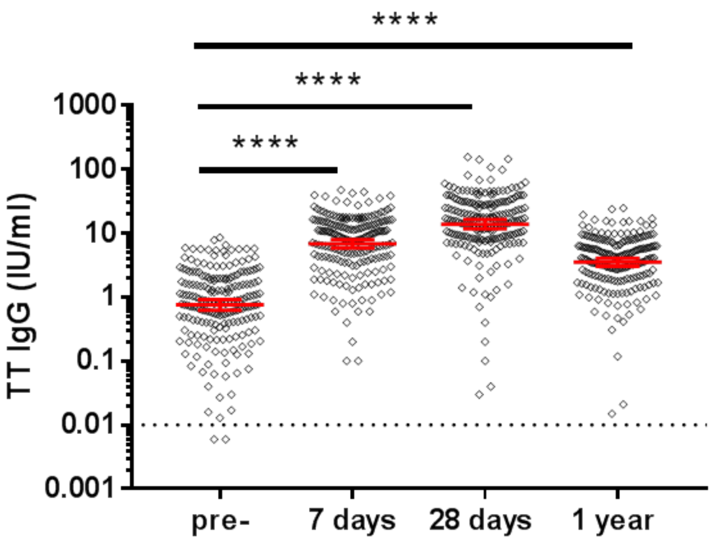

Supplementary Figure 1. TT specific IgG responses.
The lines indicate the geometric mean concentrations (GMCs) with 95% CI intervals. The different time points were compared with the repeated measured ANOVA **** ρ<0.0001.
