# Supplementary material for: Novel Intervention in the Aging Population: A Primary Meningococcal Vaccine Inducing Protective IgM Responses in Middle-Aged Adults
Source: Front Immunol. 2017 Jul 19;8:817. doi: 10.3389/fimmu.2017.00817 (PMC5515833; doi:10.3389/fimmu.2017.00817)
Supplement: Supplementary file 3 [file Image_2.PDF]

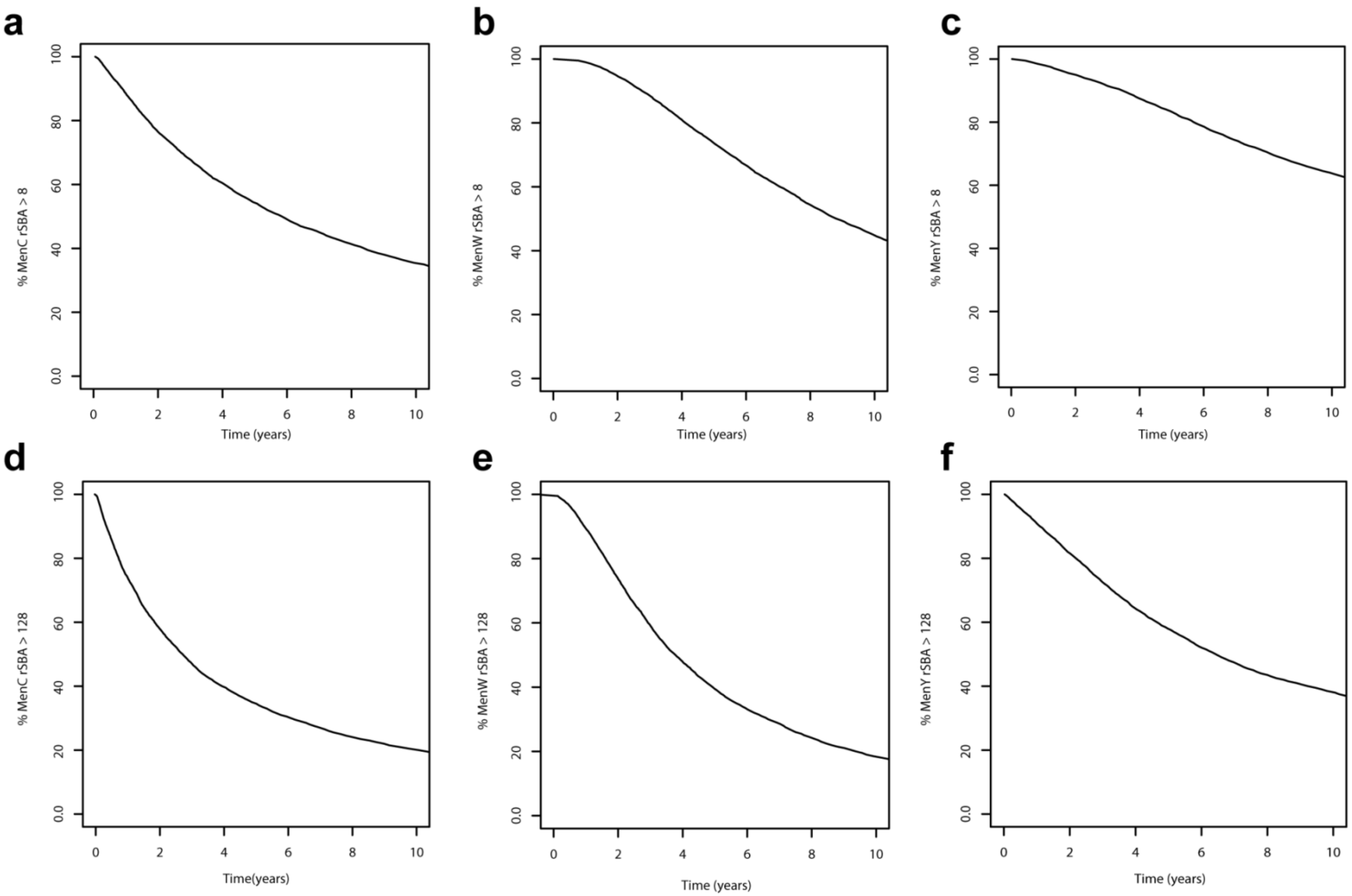

Supplementary Figure 2. Prediction of the protective rSBA titers up to 10 years post-vaccination.
The percentage of participants with an rSBA titer above 8 **(a-c)**, and above 128 **(d-f)** for MenC **(a,d)**, MenW **(b,e)**, and MenY **(c,f)** as predicted by the bi-exponential decay model.
