# Supplementary material for: Novel Intervention in the Aging Population: A Primary Meningococcal Vaccine Inducing Protective IgM Responses in Middle-Aged Adults
Source: Front Immunol. 2017 Jul 19;8:817. doi: 10.3389/fimmu.2017.00817 (PMC5515833; doi:10.3389/fimmu.2017.00817)
Supplement: Supplementary file 4 [file Image_3.PDF]

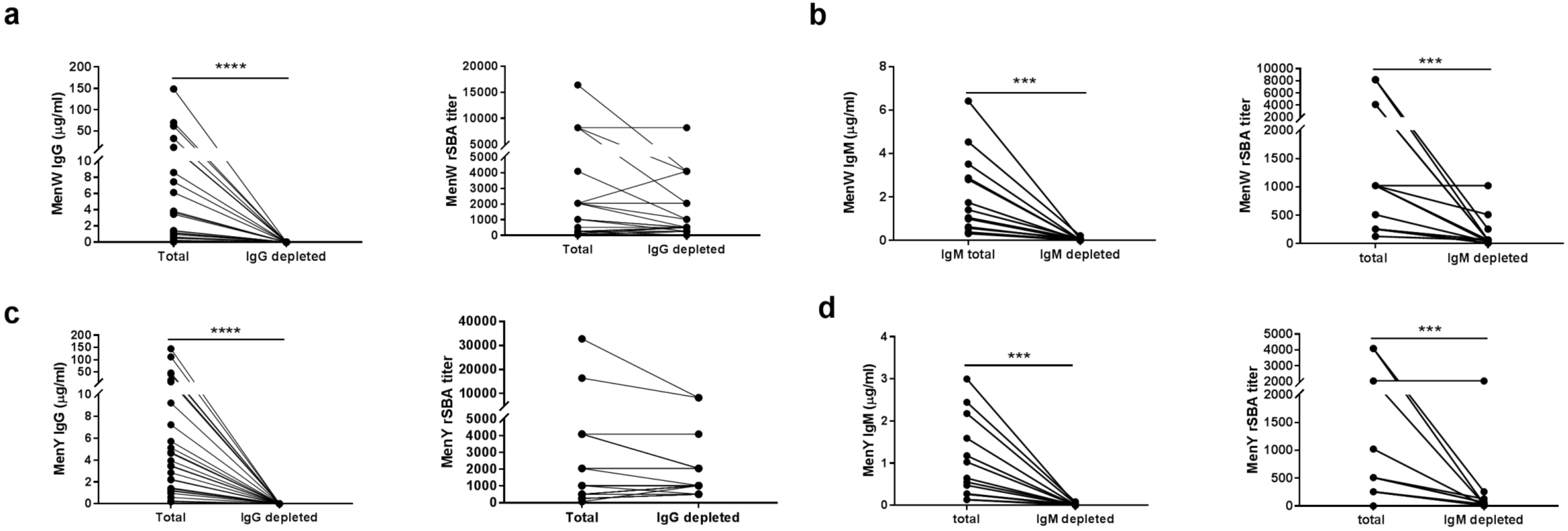

Supplementary Figure 3. Effects of IgG and IgM depletion on the post-vaccination rSBA responses.
a). MenW-PS specific IgG (left) and rSBA (right) responses after IgG removal with Gullsorb incubation. N=28 **b).** MenW-PS specific IgM (left) and rSBA (right) responses after IgM removal with anti-IgM agarose beads. N=14 **c).** MenY-PS specific IgG (left) and rSBA (right) responses after IgG removal with Gullsorb incubation. N=28 **d).** MenY-PS specific IgM (left) and rSBA (right) responses after IgM removal with anti-IgM agarose beads. N=14. The total and depleted samples were compared with the with Mann Whitney U test. *** ρ< 0.001, **** ρ< 0.0001.
