# Supplementary material for: Novel Intervention in the Aging Population: A Primary Meningococcal Vaccine Inducing Protective IgM Responses in Middle-Aged Adults
Source: Front Immunol. 2017 Jul 19;8:817. doi: 10.3389/fimmu.2017.00817 (PMC5515833; doi:10.3389/fimmu.2017.00817)
Supplement: Supplementary file 5 [file Table_1.DOCX]

**Supplementary table 1. Participant characteristics**

# Medication used more than 3 months ago and mainly consisting of corticosteroids and antibiotics.

| **Parameter** |  |
| --- | --- |
| BMI (range) | 25.5 (18.1 – 37.2) |
| CMV seropositive (number, %) | 101 (49.5%) |
| **Diseases in last year (number, %)** |  |
| Diabetes type II | 9 (4.4 %) |
| High blood pressure | 32 (15.7%) |
| Vascular diseases | 6 (2.9%) |
| Lung diseases | 7 (3.4%) |
| Rheumatic diseases | 4 (2.0%) |
| Gastro-intestinal diseases | 5 (2.5%) |
| Other diseases | 11 (5.4%) |
| No serious diseases | 146 (71.6%) |
| **Medication last 6 months (number, %)** |  |
| Medication for infections # | 15 (7.4%) |
| Cholesterol lowering medication | 26 (12.7%) |
| Diabetic medication | 8 (3.9%) |
| Blood pressure lowering medication | 38 (18.6%) |
| Immunosuppressive medication # | 3 (1.5%) |
| No medication | 143 (70.1%) |
| **Infections (number, %)** |  |
| Influenza < 4 weeks | 5 (2.5%) |
| Cold <4 weeks | 34 (16.7%) |
| No infection < 4 weeks | 168 (82.4%) |
| **Smoking (number, %)** |  |
| Cigarette smoking | 28 (13.7%) |
| Cigars, pipe smoking | 8 (3.9%) |
| No smoking | 168 (82.4%) |
| **Physical activity (numbers, %)** |  |
| Weekly or more | 141 (69.1%) |
| Less than weekly | 24 (11.8%) |
| No activity | 39 (19.1%) |
