# Supplementary material for: Novel Intervention in the Aging Population: A Primary Meningococcal Vaccine Inducing Protective IgM Responses in Middle-Aged Adults
Source: Front Immunol. 2017 Jul 19;8:817. doi: 10.3389/fimmu.2017.00817 (PMC5515833; doi:10.3389/fimmu.2017.00817)
Supplement: Supplementary file 6 [file Table_2.DOCX]

**Supplementary Table 2. Effect of age and gender on the MenCWY-PS IgG responses.**

| **Group** | **Measure** | **Time point** | **Predicting variable** | **ρ-value** | **β coefficient** | **R^2^ model** |
| --- | --- | --- | --- | --- | --- | --- |
| MenC | IgG | 7 days | **Pre- IgG** | **0.000*** | **0.421** | 0.181 |
|  |  |  | Age | 0.177 | -0.087 |  |
|  |  |  | Gender | 0.888 | -0.009 |  |
|  |  | 28 days | **Pre- IgG** | **0.000*** | **0.331** | 0.095 |
|  |  |  | Age | 0.928 | 0.006 |  |
|  |  |  | Gender | 0.860 | -0.012 |  |
|  |  | 1 year | **Pre-IgG** | **0.000*** | **0.437** | 0.175 |
|  |  |  | Age | 0.315 | 0.067 |  |
|  |  |  | Gender | 0.885 | -0.010 |  |
| MenY | IgG | 7 days | Pre- IgG | 0.693 | 0.028 | 0.008 |
|  |  |  | **Age** | **0.044*** | **-0.143** |  |
|  |  |  | Gender | 0.502 | 0.047 |  |
|  |  | 28 days | Pre- IgG | 0.549 | -0.043 | -0.011 |
|  |  |  | Age | 0.624 | -0.035 |  |
|  |  |  | Gender | 0.607 | 0.037 |  |
|  |  | 1 year | Pre-IgG | 0.885 | -0.010 | -0.012 |
|  |  |  | Age | 0.889 | 0.010 |  |
|  |  |  | Gender | 0.448 | 0.055 |  |
| MenW | IgG | 7 days | **Pre- IgG** | **0.000*** | **0.424** | 0.227 |
|  |  |  | **Age** | **0.023*** | **-0.143** |  |
|  |  |  | **Gender** | **0.007*** | **0.171** |  |
|  |  | 28 days | **Pre- IgG** | **0.000*** | **0.269** | 0.095 |
|  |  |  | Age | 0.103 | -0.110 |  |
|  |  |  | **Gender** | **0.040*** | **0.140** |  |
|  |  | 1 year | **Pre-IgG** | **0.000*** | **0.302** | 0.098 |
|  |  |  | Age | 0.380 | -0.060 |  |
|  |  |  | Gender | 0.100 | 0.114 |  |

Linear regression was performed using the log-transformed IgG and IgM concentrations. Age was included as a continuous variable. Gender. 0: females, 1: male. The effects of age and gender were adjusted for the presence of pre-vaccination immunity (Pre-IgG), since pre-IgG was perceived a confounder. Significant correlations were indicated in bold. After correction for multiple testing, a ρ value < 0.05/9 = 0.006 was considered significant. These correlations are underlined.
