# Supplementary material for: Novel Intervention in the Aging Population: A Primary Meningococcal Vaccine Inducing Protective IgM Responses in Middle-Aged Adults
Source: Front Immunol. 2017 Jul 19;8:817. doi: 10.3389/fimmu.2017.00817 (PMC5515833; doi:10.3389/fimmu.2017.00817)
Supplement: Supplementary file 7 [file Table_3.DOCX]

**Supplementary Table 3. Correlation between age and rSBA titers.**

|  | **MenC** | **MenY** | **MenW** |
| --- | --- | --- | --- |
| 28 days | **Rho = -0.239 ρ= 0.017** | Rho = -0.109 ρ= 0.281 | Rho = -0.191 ρ= 0.056 |
| 1 year | Rho = -0.071 ρ= 0.483 | Rho = -0.009 ρ= 0.927 | **Rho = -0.300 ρ= 0.002** |

The Spearman correlation was used. N=100.
