# Supplementary material for: Novel Intervention in the Aging Population: A Primary Meningococcal Vaccine Inducing Protective IgM Responses in Middle-Aged Adults
Source: Front Immunol. 2017 Jul 19;8:817. doi: 10.3389/fimmu.2017.00817 (PMC5515833; doi:10.3389/fimmu.2017.00817)
Supplement: Supplementary file 8 [file Table_4.DOCX]

**Supplementary Table 4. rSBA titers in participant with (+) and without (-) a pre-vaccination rSBA titer.**

| **Time point** | **Value** | **MenC** | | **MenW** | | **MenY** | |
| --- | --- | --- | --- | --- | --- | --- | --- |
|  |  | **-** | **+** | **-** | **+** | **-** | **+** |
| **Pre-** | **GMT [95% CI]** | 2 [2 – 2] | 49.2 [22.3 – 108.1]*** | 2 [2-2] | 128 [81.1 – 202.1]**** | 2 [2 – 2] | 193 [127 – 293.3]**** |
|  | **≥8 % [95% CI]** | 0 [0.0 – 4.6] | 85.7 [65.4 – 95.0]*** | 0 [0.0 – 4.8] | 95.87 [79.8 – 99.8]*** | 0 [0.0 – 5.0] | 100 [87.5 – 100]*** |
|  | **≥128 [95% CI]** | 0 [0.0 – 4.6] | 33.3 [17.2 – 54.6]*** | 0 [0.0 – 4.8] | 33.3 [18.0 – 53.3]*** | 0 [0.0 – 5.0] | 63 [44.2 – 78.5]*** |
| **28 days** | **GMT [95% CI]** | 1309 [776.9 – 2207] | 2261 [1122 – 4556] | 1558 [1140 – 2129] | 2656 [1573 – 4484] | 1226 [794 – 1894] | 2269 [1381 – 3730] |
|  | **Ratio 28d/pre- [95% CI]** | 654.7 [388.4 – 1104] | 46.01 [18.4 – 115.2]*** | 730.7 [512.3 – 1042] | 20.8 [10.0 – 43.12]*** | 613.2 [397.1 – 947.1] | 11.8 [6.4 – 21.6]*** |
|  | **≥8 [95% CI]** | 92.4 [84.4 – 96.5] | 100 [84.5 – 100] | 98.7 [92.9 – 99.9] | 100 [86.2 – 100] | 95.9 [88.6 – 98.9] | 100 [87.5 – 100] |
|  | **≥128 [95% CI]** | 89.9 [81.3 – 94.8] | 100 [84.5 – 100] | 96.1 [89.0 – 98.9] | 100 [86.2 – 100] | 93.2 [85.0 – 97.0] | 100 [87.5 – 100] |
| **1 year** | **GMT [95% CI]** | 84.0 [46.4 – 152.1] | 322.5 [ 124.3 - 836.9]* | 238 [157.3 – 360.2] | 994.8 [666.9 – 1484]**** | 153.3 [88.3 – 266.2] | 900.6 [556.6 – 1457]*** |
|  | **Ratio 28d/1y [95% CI]** | 15.6 [10.1 – 24.0] | 7.0 [3.3 – 14.8] | 6.3 [4.7 – 8.3] | 2.7 [1.7 – 4.3]** | 8.0 [5.6 – 11.4] | 2.5 [1.8 – 3.6]*** |
|  | **≥8 [95% CI]** | 72.2 [61.4 – 80.8] | 90.5 [71.1 – 98.3] | 92.1 [83.8 – 96.3] | 100 [86.2 – 100] | 80.8 [70.3 – 88.2] | 100 [87.5 – 100]** |
|  | **≥128 [95% CI]** | 55.7 [44.7 – 66.1] | 81 [64.0 – 94.8]* | 67.1 [55.9 – 76.6] | 95.8 [79.8 – 99.8]** | 71.2 [60 – 80.3] | 100 [87.5 – 100]** |

The Mann-Whitney U test was used to compare the GMTs, and the Chi-Square test to compare the proportion of participants above the indicated threshold between the participant with (+) and without (-) pre-vaccination rSBA titers. *ρ<0.05, **ρ<0.01, ***ρ<0.001, ****ρ<0.0001.
